# Do Health and Housing Attributes Motivate Residential Moves Among Older Chinese Adults? Evidence From an 8‑Year Follow‑up Study

**DOI:** 10.1093/geroni/igae049

**Published:** 2024-05-14

**Authors:** Ziqi Zhang

**Affiliations:** Department of Architecture, School of Design, Shanghai Jiao Tong University, Shanghai, China

**Keywords:** Health, Housing, Residential moves, Self-rated health

## Abstract

**Background and Objectives:**

Despite the widespread belief in aging-in-place as the preferred housing arrangement for older adults, they are increasingly embracing moving. The person–environment fit theory explains that environmental changes due to residential moves can pose health risks, discouraging older people from moving. However, it also suggests that moves may be suitable when living conditions no longer meet their physical needs. The correlation between older people’s health, housing, and their subsequent moving or staying actions in China remains underexplored.

**Research Design and Methods:**

Using alternative operating variables of key concepts and the China Family Panel Studies 2012–2018 data sets, this study examines the effects of health and housing status on older people’s residential moves in China. The study outlines changes in health indicators and housing characteristics during multiple moves, as well as examines the relationship between the health and housing status of older adults and moving over a relatively long period of time using both regression models with lagged explanatory variables and fixed effects binary choice models.

**Results:**

The results reveal that: (1) higher rates of subsequent moves were observed among older adults with better self-rated health, positive self-rated health changes, and no ADL impairment, but no significant associations were found between most health variables and moving; and (2) the correlation between older persons’ house ownership/type and their residential moves was significant and consistent over time, steady and lasting.

**Discussion and Implications:**

Potential mechanisms explaining the association between specific housing types and ownership statuses on moving are discussed. The findings encourage a focus on the positive benefits of moving in later life and how to provide additional housing options for older individuals.

**Translational Significance:** Gaining a deeper understanding of how health and housing conditions influence the residential moves of older individuals in relation to their preferred aging location is crucial yet remains understudied. The study reveals the stable impact of housing type and ownership, rather than health, on successive moves. Policymakers and government managers stand to benefit from comprehending the challenges faced by older adults who move their homes, allowing them to develop effective measures that support successful aging-in-place or help achieve their moving goals by identifying the factors influencing their decisions to move.

## Background and Objectives

China’s aging trajectory has been expedited by a declining birth rate and extended life expectancy (Lu & Liu, 2019), resulting in a population of 164.5 million persons older than 65 years, with 26 million surpassing 80 years in 2019 (Fang et al., 2020). The formidable challenges emanating from this aging trend are compounded by the ramifications of cross-province young-labor migration and the one-child policy has resulted in a reduction in family support (Cheng et al., 2019; Feng et al., 2013). Over the past three decades, older adults have become an increasingly mobile demographic (Bradley et al., 2008; Oswald & Rowles, 2017), inciting scholarly interest. However, knowledge gaps persist regarding the moving patterns of older Chinese individuals, despite their substantial numbers. In 2010, there were already 9.34 million moves among those aged 60 and older, a number that swelled to 13.04 million by 2015 (Gao et al., 2021; Zhang et al., 2018). As the aging population burgeons and urbanization advances, the trend of moving in older age is growing, giving rise to subgroups such as “snowbirds,” “old drifters,” and older migrant laborers.

For decades, China has predominantly relied on home-based care as its main aging strategy, with older adults living in their own or their children’s homes and receiving care from family and informal caregivers (Xing et al., 2018). In contrast, institutionalization is often linked with homelessness. In more developed areas, home- and community-based services that provide daily assistance such as cleaning, personal care, rehabilitative exercise, and counseling, have extended or supplemented home-based care (Chen & Han, 2016). Despite the reliance on home-based care, its effectiveness remains understudied (Feng et al., 2020). Generally, traditions, lower financial capacity, and limited care options have reduced preference for paid services. Consequently, unlike the well-examined housing-to-institution transition cases, China’s context, where institutionalized eldercare services or housing alternatives are not yet mainstream, primarily involves moves between dwellings. It remains unexplored whether health or housing plays a role in older people’s moves in China’s social context.

Longitudinal studies hold paramount significance in probing the interplay among demographic dynamics, residential moving (RM), and spatial configuration through the life course lens. They furnish invaluable insights into the dynamic interactions between older people and their evolving societal context (Davies & Pickles, 1985; Lekkas et al., 2017). Studies with confined follow-up periods (1 or 2 years) may not be predictive of moving intentions/actions indicated in the relatively far future (Robison & Moen, 2000). Studies combining size and duration (Choi, 1996) may overlook the intricate circuits of residential moves (Coulter et al., 2016). This study aims to employ a longitudinal national data set of considerable scale to explore the correlation between the health status and housing conditions of older adults and their subsequent moves, as well as to delineate the link between changes in health and housing among older adults across distinct moving scenarios. Leveraging available data, the study contributes to relevant literature across three facets. Firstly, it employs a nationally representative data set that includes older adults from various regions, age groups, socioeconomic strata, and family statuses, thereby enhancing the generalizability of findings. Secondly, it utilizes a longitudinal data set spanning four time segments (three intervals) for more than 8 years to investigate the association between moving, contemporaneous health, and housing characteristics, allowing the investigation of multiple moves over time. Lastly, acknowledging existing inconsistencies in key concepts that might lead to incongruous conclusions, it incorporates various conceptualizations of health and systematically investigates them across multiple time spans to ensure robust conclusions. These efforts contribute to a better understanding of the challenges faced by older movers and may guide policymakers in developing age-friendly strategies. Such strategies can either foster successful aging-in-place or facilitate RM by identifying pivotal factors influencing these decisions.

## Literature Review

### Moving in the Old Age

Aging in familiar environments nurtures a sense of home and attachment to place, thereby maintaining older adults’ identity and independence that enhance their quality of life (Peace et al., 2011; Rowles, 1983). The person–environment fit theory (Lawton & Nahemow, 1973; Lawton & Simon, 1968) underscores the significance of aging-in-place by asserting that older people’s well-being hinges on the interplay between the environmental press and their adaptive capacities. Moving-induced environmental changes disrupt residential normalcy, introducing the stress of adapting to a new setting and affecting the well-being of older adults with lower competence (Sergeant et al., 2008). Lawton’s environmental docility hypothesis further suggests that older adults with reduced physical or cognitive strength are more vulnerable to adverse life outcomes (Golant, 2011). Consequently, moving emerges as one of the most prevalent and challenging stressors for them (Löfqvist et al., 2013).

Older people’s perceptions of staying in their current residence or considering a move are intricately intertwined with various life events, such as retirement, widowhood, and disability (Cutchin, 2001). The developmental perspective of migration patterns in later life (Litwak & Longino, 1987) delineates the motivations and moving patterns stemming from various life events one may experience while growing older. It suggests that the period immediately following postretirement is characterized by a pursuit of an enhanced lifestyle, followed by stages aimed at addressing declining health by seeking familial assistance or transitioning to institutional care due to deteriorating health and limited social support. This model primarily highlights the influence of individual factors on the decision to move. An alternative perspective posits that push and pull factors can explain older adults’ dissatisfaction with their current housing and subsequent moving decisions (Walters, 2002). The categorization based on distance further suggests that short-distance moves are driven by push factors such as dissatisfaction with housing or neighborhood conditions due to deteriorating health, whereas long-distance moves are influenced by pull factors related to destination characteristics, such as cost of living or housing maintenance challenges (Bradley et al., 2008). These formulations mainly emphasize the impact of objectively defined environmental indicators. In contrast to the conventional life course model (Rossi, 1955), Golant’s (2011) theoretical model of residential normalcy posits that, alongside material adaptations, residential normalcy may lead individuals to either move in later life or remain in a setting where they feel a distinctive attachment and sense of security. The concept of “option recognition” (Peace et al., 2011) supports this perspective by acknowledging the potential variations in defining “home” in different situations.

Although the majority of the aforementioned conclusions stem from Western literature, it can be postulated that the decision to remain in one’s current residence is influenced by factors such as personal identity and social/emotional ties to the local area, as well as the potential economic costs associated with moving, which may outweigh the potential drawbacks of the substandard housing and limited accessible goods or services associated with aging. Conversely, the decision to move necessitates overcoming the loss of identity while pursuing improved personal health and the need for suitable housing or external support. This process may also prompt a reinterpretation of the concept of “home,” gradually acclimating older adults and cultivating attachments toward their new living arrangement. Changes in personal health and the compatibility between the housing environment and individual requirements, two key factors emphasized in the person–environment fit theory, appear to be the primary catalysts for moving among older adults.

### Health and Housing As Motives for Moving

Health is generally considered a crucial factor in later-life moves, especially institutionalization. Yet, the effect of health on moving exhibits variation across studies. The majority of research indicates that declining health, including physical functionality and self-perceived health status, tends to escalate the intention for or direct occurrence of moves among older adults (Choi, 1996; Crisp et al., 2013; Franco et al., 2021; Hallberg & Lagergren, 2009; Longino et al., 1991; Stoeckel & Porell, 2010). Nevertheless, certain studies suggest that declining health status may impede or fail to predict moving plans or actual moves; this causal link may only be applicable to those who are cognizant of their deteriorating health (De Jong et al., 1995).

Housing also plays a significant role in enhancing the quality of life in later years (Erickson et al., 2006). Individuals experiencing declining functional capacity are particularly susceptible to substandard housing conditions (Costa-Font et al., 2009; Houben, 2001). Although older people with higher residential satisfaction tend to stay in their current living arrangements (Speare, 1974), moving is usually perceived as a rejection of the present living environment to obtain a more functional dwelling (Fokkema et al., 1996; Hansen & Gottschalk, 2006). Housing factors seldom operate in isolation; instead, they incite a move by being perceived or actually not (re)matching the older person’s physical competence. Relevant research may examine various housing aspects such as length of residence, housing tenure, size, age, floor level, anticipated deterioration, and their relationship to moves (Bäumker et al., 2012; Chaulagain et al., 2021; Diaz-Serrano, 2006; Edmonston & Lee, 2014; Erickson et al., 2006; Fokkema & Van Wissen, 1997; Sommers & Rowell, 1992). The broader construct of residential satisfaction, encompassing housing units, social domains, community contexts, and connections, has also been explored (Andrews & Phillips, 2005; Diaz-Serrano, 2006; Diaz-Serrano & Stoyanova, 2010; Hillcoat-Nallétamby & Ogg, 2014; Kahana et al., 2003; Oh, 2003).

Older people’s moves in China are often studied with health as the outcome rather than the driving impetus. Positive associations between rural-to-urban migration, intraurban moves, and short-term migration with self-reported health status have been found (Gao et al., 2021; Hou et al., 2019; Song & Sun, 2016), although opposing viewpoints exist (Lu et al., 2020; Wang & Hu, 2019). Housing attributes exhibit a more robust influence on moving decisions than objective or self-reported health indicators (Dou & Yujun, 2015). Living conditions, such as housing size, value, and neighborhood, emerge as pivotal drivers (Liu et al., 2021). However, the findings manifest complexity due to data variations, sample characteristics, and regional dynamics. Although some argue that social security and family considerations significantly shape the moving patterns of older Chinese people within sociopolitical and economic contexts, existing knowledge suggests that the utilization of basic public health services is influenced by social networks and physical activity rather than immigration characteristics (Lin et al., 2021); a tenuous statistical link has been identified between the number of family members and older movers (Dou & Yujun, 2015). Aligned with the theoretical underpinning of the interaction between older people and the physical environment, this study will predominantly concentrate on examining the influence of health and housing factors.

## Research Design and Methods

### Data Source

This study used data from the China Family Panel Studies (CFPS), which were initiated biennially since 2010 by the China Social Science Survey Center (Institute of Social Science Survey [ISSS]). The data set employs a three-tiered implicit stratification: districts/counties, administrative villages/neighborhood committees, and family households. The initial sample consists of 16,000 households from 25 provinces/municipalities/autonomous areas. Sample loss may occur due to death, expulsion, or changes in core study participants’ status. The study primarily focused on older people (age ≥ 60, as defined by *the Law of the People’s Republic of China on Protection of the Rights and Interests of the Elderly*) and drew data from four waves: 2012, 2014, 2016, and 2018. The sample sizes for constructing models were tailored to align with specific research objectives.

### Measures

A move is usually defined as a transition from one residential address to another between two successive surveys (De Jong et al., 1995; Nowok et al., 2018; Sander & Bell, 2014). In the CFPS questionnaire, if a respondent answered “no” to the question “Is the address recorded in the last wave your current primary residence?” it signified a residential move during the preceding 2 years.

The CFPS includes a number of physical health measures. Self-rated health (SRH), self-rated health change over the past year (SRHC), and activities of daily living scores (ADL) were selected as operational health variables. Although recognizing that dichotomizing multicategorical and continuous variables entails an unavoidable loss of information in the data, the main objective was to compare the proportion of subsequent moves among older people in the “(becoming) healthy” and “(becoming) unhealthy” scenarios. Hence, certain variables were transformed into binary categories to facilitate a clearer interpretation of the outcomes. For SRH, “excellent,” “very good,” and “good” were grouped as “good,” while “fair” and “poor” were grouped as “fair/poor”; for SRHC, considering the tendency for health to deteriorate in older adults, “getting better” and “remaining the same” were combined into one category and “getting worse” was listed as a separate category. ADL was scored by measuring the ability to perform seven fundamental daily activities, including going outdoors, eating a meal, kitchen activities, using public transportation, shopping, cleaning, and laundering independently. Respondents were asked to identify activities they could not perform without assistance by selecting cards in the 2012 and 2014 waves while responding affirmatively or negatively in the 2016 and 2018 waves. A nonselected card or a “yes” response denoted the participant’s ability to complete certain daily activities, each meriting 1 point. Consequently, each older participant received an ADL score ranging from 0 to 7. Considering the ADL assessment’s connotation, where inadequate performance in any one of the daily activities signifies a considerable need for external assistance, ADL scores of 0 to 6 were grouped and coded as 0 to indicate ADL impairment, and respondents with a score of 7 coded as 1 indicating no ADL impairment.

Two variables, housing type (HT) and homeownership (HO), from CFPS in all waves were selected to characterize the housing situation. *Housing types* encompass flats, bungalows, quadrangles, villas (including townhouses), detached houses, and others. The original questionnaire entries did not provide detailed descriptions of each housing type. Flats generally refer to residential buildings with private kitchens and toilets, similar to apartments in Western countries. Bungalows typically represent self-built single-story dwellings with subpar living conditions. Detached houses generally indicate residences constructed by rural farmers on their own homesteads. *Housing ownership* comprises family members with full or partial property rights, public housing (houses provided by government/employers), government rental housing, commercial rental housing, and properties owned by others (relatives or friends). Public housing refers to housing sold to existing tenants at a price close to the construction cost after 1978, when the commodification and marketization of housing began (Zhou & Ronald, 2017). Government rental housing includes public rental housing that is provided to college graduates and specific groups facing housing difficulties, and low-rent housing, which offers rent subsidies or allocations to families meeting minimum living security standards. The variance inflation factor (VIF) and Mean VIFs suggest that there is no collinearity between housing ownership and housing type across all waves. The “flats” category of housing type and the “full ownership” category of housing ownership are used as the reference categories in the regressions.

The 2012/2014 waves also include a “housing overdensity” variable (HD), which measures whether respondents have the following living situations: “children over the age of 12 living in the same room with their parents,” “young and old family members living in the same room,” “children of the opposite sex over the age of 12 living in the same room,” “beds that are set up at night and removed during the day,” and “beds that are set up in the living room.” Respondents who indicated yes to living in one or more of the listed living situations were determined to have housing overdensity. This item was only included in the lagged regression model.

Besides, age, gender, education, marital status, self-reported relative local income, and family size served as additional control variables. “Self-reported relative local income” was measured using the following question “What do you think your personal income level in your local area,” which was asked on a five-point Likert scale ranging from “very low” to “very high.” Family size is a measure of the number of economically interconnected household members, interpreted by the CFPS guidelines.

### Statistical Analysis

The first analysis focuses on examining the effect of both health and housing variables on future moves over a 2-year period. Whether a move occurred in period *t* serves as the dependent variable, whereas health and housing variables in period *t*−1 are considered the core independent variables. Other demographic and household characteristics measured at *t*−1 are included in the models as controls. This study employs separate models for three time intervals (12–14, 14–16, 16–18), in which each of the three “health variables,” ADL, SRH, and SRHC are included in separate models, resulting in a total of nine models. Due to varying amounts of missing data across the three health variables, some of these models have different sample sizes. Binary logistic regression analyses were conducted given the dichotomous outcome. The inclusion of lagged explanatory variables helps mitigate endogeneity issues in the estimation of contemporaneous effects.

The second analysis investigates the relationship between moving homes and both health and housing status by incorporating a time effect. Graphs and descriptive statistics depict the evolution of health and housing conditions over time. Subsequently, a fixed-effects binary choice model for panel data spanning three time intervals is constructed as follows:


$$\begin{array}{lll} Relo_{it}= & \alpha_{1}Health_{it}+\alpha_{2}Housing_{it}+\beta_{1}X_{it}+\beta_{2}Z_{i}\; & +\mu_{i}+\lambda_{t}+{\;\varepsilon \;}_{it} \end{array}$$

Relo_*it*_ refers to whether the respondent moved in the 2-year period; Health_*it*_ and Housing_*it*_ are time-varying health status and housing status; X_*it*_ represents time-varying control variables, including age (log-transformed to prevent colinearity), relative income, and family size with economic ties; μ_*i*_ refers to the unobservable individual fixed effect; *Z*_*i*_ represents time-invariant control variables, including gender, educational attainment (the educational attainment of older adults is assumed not to change again), marital status, and “hukou” type (the unique household registration system in China that classifies the population into either rural or urban households); λ_*t*_ accounts for the fixed effect of time, and ε_*it*_ represents the random error term. The fixed effects model mitigates the endogeneity problem by eliminating or controlling for individual heterogeneous effects that remain constant over time, that is, both µ_*i*_ and *Z*_*i*_ are eliminated. The model also offers the advantage of a larger sample size by incorporating data from the concluding point of the 2014, 2016, and 2018 waves (excluding the 2012 wave, as it lacked moving status), thus facilitating a more accurate estimation. Heteroskedasticity robust standard errors are utilized and covariance is checked using VIFs. Stata 16 was used to conduct all statistical analyses.

## Results

### The Effect of Health and Housing Status on Older Adults’ Moves

Table 1 displays the proportion of older adults with specific health conditions who experience RM in the subsequent one to three waves (2 to 6 years). It also shows the correlation between baseline health status and the occurrence of a move. The proportion of older adults who experienced moves ranges from 5% to 10% of the total sample. Older adults with better SRH (“good”), positive SRHC (“better/remaining the same”), or better objective health (“no ADL impairment”) generally have higher rates of moving, except for ADL12 to RM16 and SRH16 to RM18. However, only the change in SRHC12/14 is significantly associated with moving in 2014/2016, whereas no significant associations were found between other health variables and moving.

**Table 1.** Health Status in *t* Period and the Moving Status Between *t* and *t* + 1

Variable	RM14		RM16		RM18	
	%	*X* ^2^	%	*X* ^2^	%	*X* ^2^
SRH12		3.4971^+^		0.2484		1.0467
Good	3.98		3.89		5.47	
Fair/Poor	2.94		3.55		4.64	
SRHC12		6.0678^*^		2.8300^+^		2.3399
Better/remaining the same	4.07		4.24%		5.61	
Getting worse	2.71		3.13%		4.38	
ADL12		3.6211^+^		0.2084		1.6993
No ADL impairment	3.60		3.66		5.20	
ADL impaired	2.02		4.15		3.37	
SRH14				0.7848		0.3437
Good			4.00		4.16	
Fair/Poor			3.49		4.57	
SRHC14				5.7001^*^		1.8663
Better/remaining the same			4.41		4.79	
Getting worse			3.05		3.84	
ADL14				0.0272		0.0742
No ADL impairment			3.78		4.38	
ADL impaired			3.62		4.05	
SRH16						1.4695
Good					3.91	
Fair/Poor					4.61	
SRHC16						0.3228
Better/remaining the same					4.41	
Getting worse					4.08	
ADL16						0.2795
No ADL impairment					4.33	
ADL impaired					3.94	

*Notes*: ADL = activities of daily living; RM = residential moving (happened or not); SRH = self-rated health; SRHC = self-rated health changes.

^*^ *p* < .05. ^+^*p* < .1.

Concerning housing situations (shown in Table 2), older adults living in (commercial) rental housing tend to have subsequent moving rates ranging from 10% to 50%. In certain waves, a greater proportion of subsequent moves is observed for government rental housing, properties owned by others, and public housing. In contrast, the fluctuations in moving rates based on HT are inconsistent over time. Overall, both HO and HT are significantly associated with moving within the next 2 to 6 years, suggesting that the effects of HO and HT are likely to have enduring consequences on moving over the long term.

**Table 2.** Housing Status in *t* Period and the Moving Status Between *t* and *t* + 1

Variable	RM14		RM16		RM18	
	%	*X* ^2^	%	*X* ^2^	%	*X* ^2^
HO12		196.5695^***^		39.2244^***^		51.7171^***^
Full ownership	2.57		3.16		4.72	
Partial ownership	4.00		0.00		6.06	
Public housing	3.13		4.55		6.25	
Government rental housing	13.58		11.54		4.17	
Rental housing	44.83		10.53		43.75	
Other people’s properties	4.61		9.52		4.26	
Others	7.91		9.45		6.48	
HT12		20.6266^***^		18.0648^**^		
Flats	5.26		4.27		7.73	14.2215^*^
Bungalows	2.45		2.20		5.06	
Quadrangles	1.99		6.19		2.02	
Villas	0.00		0.00		0.00	
Detached houses	4.41		5.53		4.34	
Others	2.33		3.93		3.15	
HD12		0.8022		2.3567		0.4961
With difficulties	4.17		2.08		5.95	
No difficulties	3.32		3.86		4.94	
HO14				83.8663^***^		41.931^***^
Full ownership			3.17		4.09	
Partial ownership			0.00		3.66	
Public housing			0.00		5.56	
Government rental housing			12.31		8.51	
Rental housing			25.00		35.29	
Other people’s properties			10.34		4.73	
Others			8.57		4.71	
HT14				56.9184^***^		22.5004^***^
Flats			5.08		6.84	
Bungalows			2.84		3.59	
Quadrangles			4.62		0.98	
Villas			17.05		10.77	
Detached houses			3.98		3.55	
Others			1.71		4.68	
HD14				1.5704		0.0933
With difficulties			2.69		4.66	
No difficulties			3.88		4.31	
HO16						296.8588^***^
Full ownership					3.29	
Partial ownership					5.56	
Public housing					11.11	
Government rental housing					11.11	
Rental housing					48.15	
Other people’s properties					4.05	
Others					12.40	
HT16						43.6057^***^
Flats					8.13	
Bungalows					3.36	
Quadrangles					2.10	
Villas					0.00	
Detached houses					3.30	
Others					4.44	

*Notes*: HD = Housing difficulties; HO = Housing ownership; HT = Housing type; RM = Residential moving (happened or not).

^***^ *p* < .001. ^**^p < .01. ^*^p < .05.

All lagged regression models demonstrate statistical significance (Prob > chi^2^ = 0.000), as shown in Table 3. The table displays the unstandardized regression coefficients. Except for individuals reporting worsened health in the “14–16 interval” model showing significantly reduced odds of moving in the next 2 years compared to those with stable or improved health, the health variables in the “12–14 interval” model and “16–18 interval” model indicate no significance at a 5% significance level. The association between housing variables and odds of moving was consistently robust across all three time intervals, albeit with slight variations in which housing variables were found to be significantly associated. Overall, individuals living in public housing, government/commercial rental housing, and other people’s properties are more likely to move compared to those with full homeownership. Renters have a moving rate 2 to 29 times higher than those with full ownership. Those residing in bungalows, quadrangles, and other housing styles are 0.3 to 0.6 times more likely to move than those residing in flats, whereas villa residents have a higher likelihood of moving. Housing overdensity was not found to be associated with odds of moving in any of the time intervals.

**Table 3.** Relationship Between Health, Housing Status, and Actual Moves During Each Time Interval

Variable	Item	12–14 interval Y = RM14			14–16 interval Y = RM16			16–18 interval Y = RM18		
		Model 1-1 H = SRH12 (*n* = 4,333)	Model 1-2 H = SRHC12 (*n* = 4,333)	Model 1-3 H = ADL12 (*n* = 4,327)	Model 2-1 H = SRH14 (*n* = 4,312)	Model 2-2 H = SRHC14 (*n* = 4,312)	Model 2-3 H = ADL14 (*n* = 4,312)	Model 3-1 H = SRH16 (*n* = 5,337)	Model 3-2 H = SRHC16 (*n* = 5,337)	Model 3-3 H = ADL16 (*n* = 5,337)
		Coef. (*SE*)	Coef. (*SE*)	Coef. (*SE*)	Coef. (*SE*)	Coef. (*SE*)	Coef. (*SE*)	Coef. (*SE*)	Coef. (*SE*)	Coef. (*SE*)
SRH (good)	Fair/Poor	−0.261 (0.176)	—	—	−0.177 (0.164)	—	—	0.102 (0.148)	—	—
SRHC (better/remaining the same)	Worse	—	−0.296^+^ (0.178)	—	—	−0.095^*^ (0.042)	—		0.006 (0.148)	—
ADL (unimpaired)	Impaired	—	—	−0.530 (0.331)	—	—	0.046 (0.270)	—	—	−0.110 (0.197)
Housing ownership (full ownership)	Partial ownership	0.427 (0.433)	0.413 (0.434)	−0.427 (0.433)	0 (empty)	0 (empty)	0 (empty)	0.383 (0.433)	0.387 (0.433)	0.390 (0.433)
	Public housing	0.142 (1.029)	0.169 (1.029)	0.155 (1.029)	0 (empty)	0 (empty)	0 (empty)	1.056^*^ (0.452)	1.052^*^ (0.452)	1.050^*^ (0.452)
	Government rental housing	1.838^***^ (0.357)	1.802^***^ (0.357)	1.838^***^ (0.357)	1.452^***^ (0.403)	1.440^***^ (0.402)	1.436^***^ (0.403)	0.962^+^ (0.546)	0.955^+^ (0.546)	0.953^+^ (0.546)
	Rental housing	3.373^***^ (0.411)	3.356^***^ (0.413)	3.415^***^ (0.410)	2.212^***^ (0.495)	2.217^***^ (0.497)	2.223^***^ (0.496)	2.944^***^ (0.283)	2.952^***^ (0.283)	2.946^***^ (0.283)
	Other people’s properties	0.495 (0.408)	0.453 (0.407)	0.455 (0.407)	1.166^***^ (0.262)	1.161^***^ (0.262)	1.158^***^ (0.261)	0.203 (0.357)	0.200 (0.357)	0.199 (0.357)
	Others	1.216^***^ (0.305)	1.220^***^ (0.305)	1.232^***^ (0.305)	0.993^**^ (0.369)	0.969^**^ (0.370)	0.997^**^ (0.369)	1.412^***^ (0.284)	1.421^***^ (0.284)	1.424^***^ (0.284)
Housing type (flats)	Bungalows	−0.501^*^ (0.230)	−0.481^*^ (0.230)	−0.486^*^ (0.230)	−0.347 (0.215)	−0.309 (0.216)	−0.348 (0.215)	−0.712^***^ (0.182)	−0.716^***^ (0.182)	−0.708^***^ (0.182)
	Quadrangles	−.576 (0.617)	−0.533 (0.617)	−0.547 (0.620)	0.409 (0.460)	0.442 (0.461)	0.401 (0.461)	−1.135^+^ (0.603)	−1.138^+^ (0.603)	−1.119^+^ (0.604)
	Villas	0 (empty)	0 (empty)	0 (empty)	1.748^***^ (0.339)	1.759^***^ (0.340)	1.727^***^ (0.339)	0 (empty)	0 (empty)	0 (empty)
	Detached houses	0.178 (0.236)	0.179 (0.236)	0.172 (0.236)	0.071 (0.237)	0.092 (0.238)	0.063 (0.237)	−0.671^**^ (0.212)	−0.671^**^ (0.212)	−0.669^**^ (0.212)
	Others	−0.626^+^ (0.323)	−0.615^+^ (0.323)	−0.623^+^ (0.322)	−0.999^*^ (0.418)	−0.984^*^ (0.418)	−1.018^*^ (0.418)	−0.481 (0.331)	−0.482 (0.331)	−0.478 (0.331)
Housing difficulties		−0.121 (0.226)	−0.105 (0.225)	−0.117 (0.225)	0.121 (0.207)	0.126 (0.206)	0.118 (0.207)	—	—	—

*Notes*: ADL = activities of daily living; RM = Residential moves (happened or not); SRH = self-rated health; SRHC = self-rated health changes. Demographic variables have been controlled.

The content in parentheses in the first column represents the reference category of the variable.

^***^ *p* < .001. ^**^p < .01. ^*^p < .05. ^+^*p* < .1.

### Trajectories of Health and Housing Change Among Older Adults With Multiple Moves

**Figure 1.**
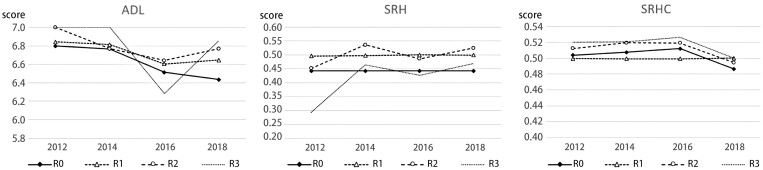

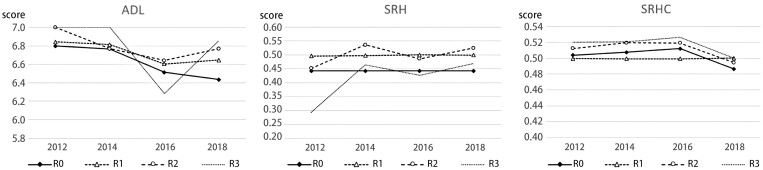
Health trends during multiple moves. ADL = activities of daily living; SRH = self-rated health; SRHC = self-rated health changes. R0 = older adults who have not moved; R1 = older adults who moved once; R2 = older adults who moved twice; R3 = older adults who moved three times.

The number of older adults who have not moved (R0), moved once (R1), moved twice (R2), or moved three times (R3) between 2012 and 2018 is 2,820, 274, 32, and 7, respectively. Given the small sample size for those with multiple moves, conclusions should be interpreted with caution. The four sample groups share similar age features (R0: 65.84 ± 5.03, R1: 66.16 ± 5.43, R2: 65.63 ± 5.17, and R3: 64 ± 5.39). Figure 1 illustrates trends in ADL, SRH, and SRHC among older adults over three time periods. The results highlight that movers exhibited higher baseline ADL scores than nonmovers. Those who did not move or moved once had little to no change in SRH, although the latter scores were higher than the former. Participants that moved twice or three times showed a fluctuating trend of increasing SRH. All cases demonstrate more positive SRHC ratings, except for those that moved once; they also had lower initial SRHC than nonmovers. Nevertheless, all movers ultimately score higher than nonmovers on all health metrics.

**Figure 2.**
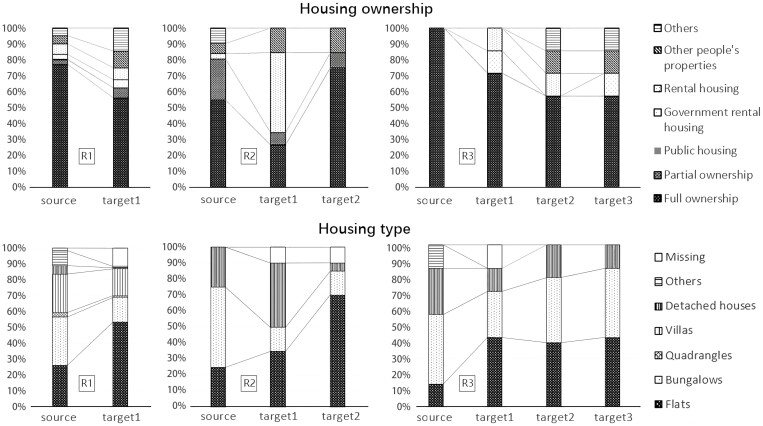

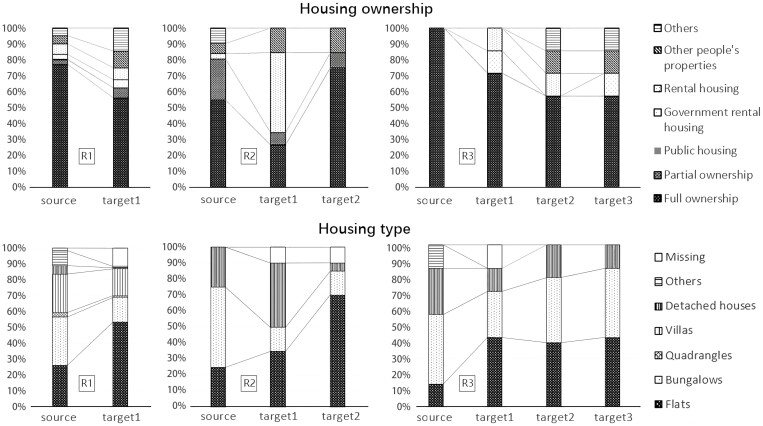
Trends of homeownership and housing type during multiple moves. R0 = older adults who have not moved; R1 = older adults who moved once; R2 = older adults who moved twice; R3 = older adults who moved three times.

Regarding housing ownership, the proportion of full housing ownership decreases among those who moved once, whereas commercial rental housing remains unchanged, and other ownership types increase. For participants who moved twice, the proportion of full ownership initially drops but subsequently exhibits an upward trend. Simultaneously, the proportion of commercial rental housing sees a substantial increase during the first move and subsequently experiences a notable decrease after the second move. The proportion of government rental housing also declines after the second move. For three-time movers, full ownership decreases in line with the number of moves, and commercial rental housing is solely observed during the transitional phase (in targets 1 and 2). In terms of housing types, the preference for flats generally increases from the initial state, whereas the proportion of bungalows, quadrangles, and villas decreases among those who moved once or twice. In the observed period, older movers ultimately tend to favor flats, bungalows, and detached houses as their preferred housing types (Figure 2).

### Longitudinal Examination of the Impact of Health and Housing Conditions on Subsequent Moves

**Figure 3.**
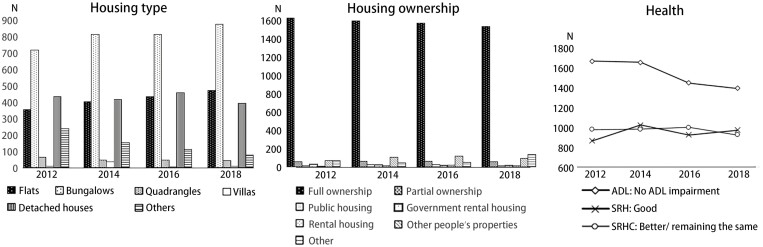

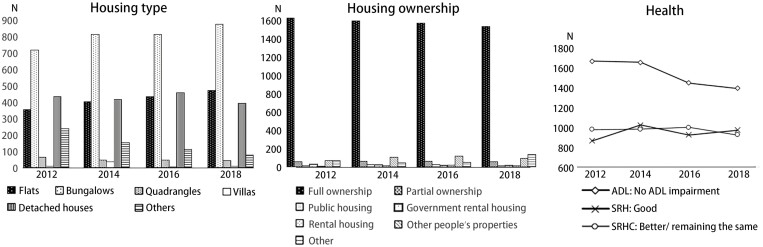
Patterns of housing/health change across the entire sample from 2012 to 2018 waves. ADL = activities of daily living; SRH = self-rated health; SRHC = self-rated health changes.

Following sample matching, the cohort consists of 1,871 older participants who participated in all three intervals, with no missing data on the pivotal housing and health variables. Figure 3 below illustrates the variations in housing type and housing ownership status over the observation period. An evident pattern emerges, reflecting an overall upward trajectory in housing types associated with flats and bungalows, accompanied by a marginal dip in homeownership status among those residing in owned properties. In terms of health, both self-assessed health measures (SRH and SRHC) exhibit a fluctuating trend, in contrast to the ADL scores, which demonstrate a pronounced and consistent decline among individuals with unimpaired ADLs.

The validation process of the fixed effects binary choice model for panel data spanning four waves, using SRH as the health variable, is illustrated as an example. The choice was first between fixed effects and mixed regression. The Hausman test strongly rejected the original hypothesis of mixed regression and suggested using a fixed effects model. The second choice, between random effects and mixed regression models, showed that the original hypothesis could not be rejected based on the LR test in random effects panel logit estimation (*p* = .078). After examining the numerical integration robustness of the random effects panel logit, all relative differences did not exceed 10^−4^, confirming the robustness of the results. Finally, the choice between fixed effects and random effects models was determined using the Hausman test, which significantly (Prob > chi^2^ = 0.000) rejected the random effects hypothesis, leading to the use of the fixed effects model. The same validation process was applied to the other two models, using different health variables. All fixed-effects binary choice models are statistically significant (Prob > chi^2^ = 0.000). As shown in Table 4, housing ownership and type are more strongly associated with the frequency of moving than objective or subjective health. Specifically, non-full ownership of property is positively associated with moves compared to full ownership of property rights; bungalows and detached houses are negatively associated with moving in comparison to flats.

**Table 4.** Results of Fixed Effects Binary Choice Models Across Three Time Intervals: Relationship Between Health, Housing Status, and Moving

Variable	Item	Health = ADL (impaired)	Health = SRH (fair/poor)	Health = SRHC (worse)
		Coef. (*SE*)	Coef. (*SE*)	Coef. (*SE*)
Health		0.475 (0.3966)	−0.142 (0.2783)	0.103 (0.2501)
Housing ownership (full ownership)	Partial ownership	3.279^**^ (0.9768)	3.071^**^ (0.9595)	3.106^**^ (0.9506)
	Public housing	3.133^*^ (1.2690)	3.033^*^ (1.2699)	3.088^*^ (1.2780)
	Government rental housing	1.439^*^ (0.6527)	1.437^*^ (0.6531)	1.420^*^ (0.6573)
	Rental housing	1.498^**^ (0.5615)	1.535^**^ (0.5637)	1.517^**^(0.5625)
	Other people’s properties	0.602 (0.4196)	0.645 (0.4174)	0.665 (0.4173)
	Others	1.899^***^ (0.4222)	1.904^***^ (0.4237)	1.883^***^ (0.4228)
Housing type (flats)	Bungalows	-2.190^***^ (0.4142)	−2.154^***^ (0.4113)	−2.140^***^ (0.4125)
	Quadrangles	−0.920 (0.8859)	−1.059 (0.8815)	−0.955 (0.8739)
	Villas	−15.754 (850.1548)	−15.770 (836.82)	−15.806 (842.05)
	Detached houses	−1.549^**^ (0.4903)	−1.560^**^ (0.4896)	−1.565^**^ (0.4913)
	Others	−2.760^***^ (0.7414)	−2.693^***^ (0.7513)	−2.715^***^ (0.7496)
Ln(age)		54.984 (50.2147)	59.738 (48.52)	56.512 (49.03)
Family size		0.024 (0.1558)	0.011 (0.1559)	0.0132 (0.1553)
Relative Income (lower)	Averaged	0.015 (0.3592)	0.030 (0.2861)	0.0174 (0.2863)
	Higher	0.423 (0.3576)	0.455 (0.3564)	0.4340 (0.3574)
Time effect	2016	−1.88 (1.4600)	−2.045 (1.4105)	−1.941 (1.427)
	2018	−3.462 (2.8271)	−3.789 (2.7306)	−3.592 (2.763)
LR chi^2^		111.58	110.08	109.99
Log likelihood		−148.9292	−150.08649	−150.13133

*Notes*: ADL = activities of daily living; SRH = self-rated health; SRHC = self-rated health changes. The content in parentheses in the first column represents the reference category of the variable.

^***^ *p* < .001. ^**^p < .01. ^*^p < .05.

## Discussion and Implications

Studies examining RM among older adults, spanning diverse cultural contexts and developmental theories, have traditionally suggested that declining health may prompt moves, a natural progression as one ages (Litwak & Longino, 1987; Miller et al., 1999; Scheibl et al., 2019). Previous research consistently highlights the predictive role of limitations in activities of daily living (ADL) and self-assessment of health status in older adults’ moves to new dwellings or institutions (De Jong et al., 1995; Granbom et al., 2014; Luppa et al., 2009; Miller & Weissert, 2000). However, this study not only fails to establish a consistent association between the three health measures examined and moving but also contradicts the hypothesis of declining health among older movers (Lubbers & Gijsberts, 2019). Instead, it reveals a positive correlation between better health and movement: older adults with better self-rated health, positive self-rated health changes, and no ADL impairment generally have higher rates of moving. Our findings to a certain extent align with the applicability of the Healthy Migrant Hypothesis in the Chinese context and internal migration (a move in this study), which suggests that healthier older adults are more likely to move (Lu & Qin, 2014; Tong & Piotrowski, 2012; Yi et al., 2019). Nonetheless, the past health status of older adults does not appear as a significant and consistent predictor of their decision to move. Upon further examination of the interpretation of the Healthy Migrant Hypothesis in previous studies and the specific population to which it pertains, it primarily applies to moving among people engaged in low-skilled labor from rural to urban areas. In this scenario, only individuals with a high physical capacity for intense labor can secure stable incomes in cities, rendering health selection a more influential factor. This phenomenon might be less applicable to older adults due to the complexity of their motivations and means to move, as employment is no longer the primary driving force for their moves. A previous study has demonstrated the joint influence of health and age on moving decisions (Wang & Zhou, 2018). In the Chinese context, better health, rather than declining health is associated with moving. This may be attributed to inadequate provision of public services for older adults and low acceptance of institutional care. When the health of older adults deteriorates, care and housing options are limited, including home-based care and institutional treatment. The former is typically an arrangement of mutual care, with older people contributing to the care of the third generation while receiving care from their children, rather than a one-way reception of care. This potentially places demands on older people’s physical capacity (Gao et al., 2021). The latter typically occurs in cases of extremely poor health, when hospitalization is seldom considered a choice, but rather an emergency. In essence, even when older adults perceive a decline in their health, they have nowhere to move. Due to health selectivity, the involuntary staying-put of older adults may be misinterpreted as a choice to “age in place.” They may have to tolerate inappropriate housing and living situations or employ different tactics to “adapt” to their current surroundings.

This investigation underscores the consistent influence of past housing conditions on moving. Existing research has distinctly outlined varying moving patterns between homeowners and tenants (Abramsson & Andersson, 2012). Homeownership directly affects moves, with tenants exhibiting a higher likelihood of moving, particularly among older adults (Abramsson & Andersson, 2016; Hansen & Gottschalk, 2006). Echoing this, our study reveals a higher proportion of subsequent moves among older adults residing in (commercial) rental housing. During certain time periods, other non-ownership housing types, including government rental housing, other people’s property, and public housing, also exhibit a higher proportion of subsequent moves. In contrast, in the multiple-move scenario, the proportion of older adults with full ownership declines among those who have made between one and three moves during the research period. This reduction is temporary in the case of two-time movers; it recovers after the initial period, indicating a housing transition tendency. It is imperative to acknowledge that the sample involving a single move may not offer a comprehensive understanding of the housing transition process due to the limited duration of move observation. In scenarios involving two or three moves, the small sample size restricts the drawing of conclusive interpretations. Research on the rental status of older adults is relatively scarce (Roy et al., 2018), and it remains unclear whether the frequent moves prompted by non-ownership housing, along with the establishment and disruption of emotional attachments to one’s home or the concept of “home,” influence residential decisions or increase the frequency of moves.

As demonstrated by prior research, housing type also affects RM (Abramsson & Andersson, 2016). For instance, a Swedish survey finds that 80% of older adults moved from single-family houses to apartments, rather than moving from a rental property to an owner-occupied residence that required more maintenance (Danermark & Ekström, 1993). A Danish study reveals that individuals living in extremely small dwellings are more likely to consider moving (Hansen & Gottschalk, 2006). Conversely, research from the United States suggests that apartment dwellers are more inclined to move within the same community compared to house dwellers (Granbom et al., 2019). Our study, based on limited data, indicates that older adults living in bungalows and detached houses are considerably less inclined to move than those living in flats. Furthermore, housing overdensity does not have a significant effect on moving. Although not explicitly described, bungalows and detached houses generally denote self-constructed houses in rural or suburban areas, which may lack official property rights, thereby limiting their entry into the housing market. The lack of a housing over-density effect on moving may also be explained by financial constraints, as some older adults may not have the means to move. This is corroborated by our examination of the link between relative income and independent moves, which reveals that in several data waves, individuals with self-reported middle income have greater odds of moving than those with lower incomes. Additionally, cultural factors influence housing preferences. In Nordic countries, for instance, people may prioritize the independence of older people, while in China, many older adults prefer to live with their children and relatives. Also, in China, older adults may not be presented with a wide range of housing options that cater to their health status. When they perceive less control over their surroundings, they often consolidate their living spaces, such as combining living rooms, bedrooms, or dining rooms into one room.

Our findings underscore that, for older adults, moving is often contingent upon specific health and financial prerequisites, rather than being a mere choice. Various factors, including difficulty in selling homes or financial limitations that hinder the affordability of alternative housing or residential care, can constrain the options available for moves. Available choices within their functional context, coupled with their awareness, motivation, ability, and confidence to explore these opportunities, play a crucial role in determining their emotional adjustment to their current living situation (Golant, 2011). Although intentions and capacities are not novel concepts in migration research, dissecting them separately provides deeper insights into the underlying reasons for either opting to move or deciding to stay. In the framework of the “aspiration/capacity model” (Carling, 2002), mobility is anticipated to occur when both the aspiration and the capacity to move are present—an active decision driven by a combination of personal circumstances and environmental factors. Voluntary immobility (i.e., having the capacity but not the desire to move), on the other hand, is the “real” aging-in-place. However, our study may indicate that involuntary immobility (i.e., having the aspiration to move but being unable to do so) among the population of older adults in China might be more prevalent than previously believed. This potentially adds a disquieting facet to the conventional view of moving in later life as a substantial risk versus the prevalent legitimacy of aging-in-place. In the “involuntary immobility” case, older adults may be more vulnerable to health risks and housing mismatches. In this investigation though, we were not able to distinguish between voluntary and involuntary moves.

In addition, housing trends reveal that in China, economic development and a real estate boom may have significantly influenced the types of housing and ownership status in this study. The development of China’s housing sector is generally considered to have occurred in three distinct phases: the Prereform Welfare Housing Sector (1949–1978), Dual-Track Housing Reform (1978–1998), and Postreform Institutional Development (since 1998; Chen & Han, 2015). The time period focused on in this study falls within the postreform market-driven phase, considered a process of neoliberal reforms. During this phase, urban housing shifted from being primarily owned and constructed by state-owned enterprises or organizations to a gradual marketization under “authoritarian control” (Wu, 2008), resulting in full private ownership of housing becoming the predominant tenure in the city (Wang et al., 2012). More relevant to the study’s time frame is the fact that China is undergoing a phase of rapid urbanization. Under this phase, China’s rural labor force continues to flock to cities (Zhang et al., 2020). Urban rental pressures led to a significant influx of rural laborers into substandard bungalows within urban villages. Consequently, the migration of these younger and middle-aged laborers has triggered the moves of older family members, who take on caregiving roles for the third generation. This phenomenon has resulted in a marginal decrease in the prevalence of full homeownership, paralleled by an upswing in bungalow dwellings. Concurrently, urbanization efforts have entailed the acquisition of farmers’ homesteads, and the farmers are often compensated with “moving upstairs” offerings—namely, flats. This might account for the progressive rise in the number of individuals residing in apartments over this study’s 8-year observation period.

Even though additional demographic factors (e.g., age, gender, marital status) were not the focus of this study, some things can be discerned from the findings. Multiple studies have shown that residential moves rise with retirement, particularly among individuals who have experienced the loss of a spouse (Danermark & Ekström, 1993; Robison & Moen, 2000). However, in this study, when examining the marital status of those who had moved 0 to 3 times, the proportions of those with or without partners (divorced, widowed) are found to be nearly equivalent across moving groups. Age and family size also exhibit notable effects. Larger family size and older age (>75 years) were found to be associated with decreased odds of moving. However, this finding was not present in all data waves.

## Conclusion

This study delves into the role of two significant factors, health and housing, in later life moves. The findings underscore the profound impact of housing type and ownership on moving and identify barriers to moving faced by older individuals, including health and financial constraints. It also incorporates a cultural comparison to augment the existing body of knowledge. The research suggests that despite the implicit encouragement of aging-in-place in China’s “building a home-based, community-relied, and institutions-supplemented eldercare service system” policy, which was enacted in 2016, it is necessary to note older people’s intentions and ability to move, and their staying-put potentially due to health or economic constraints. Possible supportive measures that could be implemented to address these challenges include: (1) providing intermediate housing solutions for older adults with declining health, bridging the gap between family and hospital care; (2) offering housing improvement services and basic medical care to older adults who are unable to move; and (3) bolstering support for intergenerational care involving the third generation. An illustrative instance of addressing the second aspect is evident in current initiatives in Shanghai, where door-to-door services are being extended and older adults’ homes are being transformed into suitable “wards,” enabling those who cannot move to access professional services that align with their current health status in their existing location.

This study has a number of limitations. First, the use of a multiyear tracking data set resulted in a decrease in sample size in subsequent data waves. To address this issue, future studies can obtain more precise estimates by employing inverse probability weighting. Second, the relatively limited sample size for individuals who underwent multiple moves may undermine the reliability of the findings specific to this group. Third, the available data lacked the capacity for an exhaustive exploration of the underlying rationales that may shape older adults’ decision-making process regarding moving. Further, the data do not allow for determining the evolving significance of place attachment and the concept of home during the process of moving. These aspects hold the potential to enrich the global discourse on the matter. Additionally, although this study spans a considerable timeframe, the inclusion of more recent and additional data waves would have allowed for a more comprehensive understanding of moving across a larger portion of the older adults’ lifespan.

## Data Availability

The data that support the findings of this study are available from the Institute of Social Science Survey (ISSS), Peking University (https://www.isss.pku.edu.cn/) with permission.
